# Dial-In Synthesis of ‘Polymer Opal’ Core–Interlayer–Shell Composite Nanoparticles

**DOI:** 10.3390/polym15173507

**Published:** 2023-08-22

**Authors:** Giselle Rosetta, Line Macaire, Mike Butters, Chris E. Finlayson

**Affiliations:** 1 Department of Physics, Prifysgol Aberystwyth University, Aberystwyth SY23 3BZ, UK; 2 Varichem Co., Ltd., Brynmawr, Blaenau Gwent, Wales NP23 4BX, UK; 3 Minton Treharne & Davies, Coryton, Cardiff CF14 7HY, UK; mike.butters@minton.co.uk

**Keywords:** polymer composites, emulsion polymerization, core-shell nanoparticles, ‘dial-in’ processing, process engineering

## Abstract

The emulsion polymerization process via which core–interlayer–shell polymer nanoparticles are synthesized is engineered to offer a crucial control of the eventual size and monodispersity of the polystyrene (PS) cores. We examine the role of key experimental parameters, optimizing the temperature, reactant purity, and agitation (stirring) rate. The subsequent addition of a poly(methyl-methacrylate) (PMMA) grafting layer and a poly(ethyl-acrylate) (PEA) shell layer produces composite particles, which are shear-orderable into opaline films, known as ‘polymer opals’. We thus demonstrate pathways toward a ‘dial-in’ process, where the time taken to obtain the target core size is mapped to the expected resultant structural color. At reaction temperatures of 60 and 70 °C, viable conditions are found where all syntheses give an excellent level of monodispersity (polydispersity index < 0.02), suitable for interlayer and shell growth. These reports may be readily applied to a wider industrial scale fabrication pipeline for these polymeric photonic materials.

## 1. Introduction

**Figure 1:**
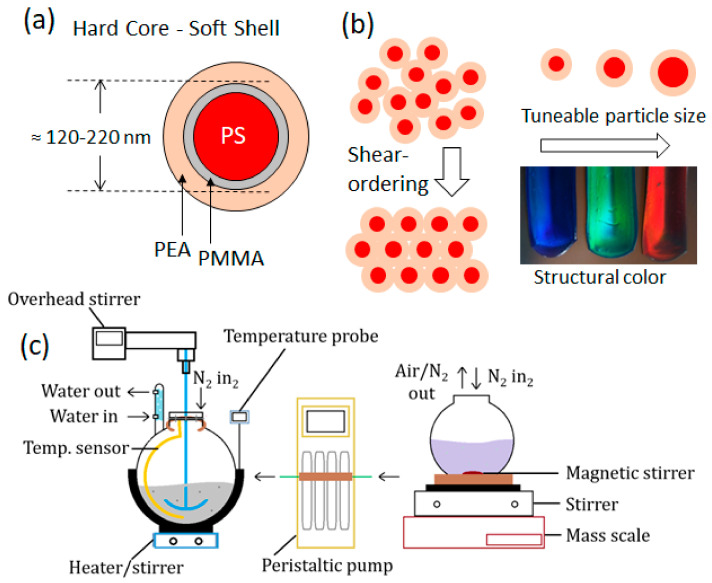
(**a**) The core–interlayer–shell structure of polymer opal nanoparticles, with layers to scale for particle diameters 100–350 nm. The constituent polymers corresponding to each layer are shown. (**b**) The shells of these monodisperse particles are deformed as order is induced using melt-shear techniques, where the polystyrene cores are fixed in the solvent-free matrix of poly(ethyl-acrylate) (**left**). The desired opaline color in the end product can be ‘dialed in’ by control of particle size (**right**). (**c**) The polymer opal nanoparticle growth apparatus, with the primary reaction vessel, peristaltic pump, and secondary monomer solution vessel shown.

**Figure A1:**
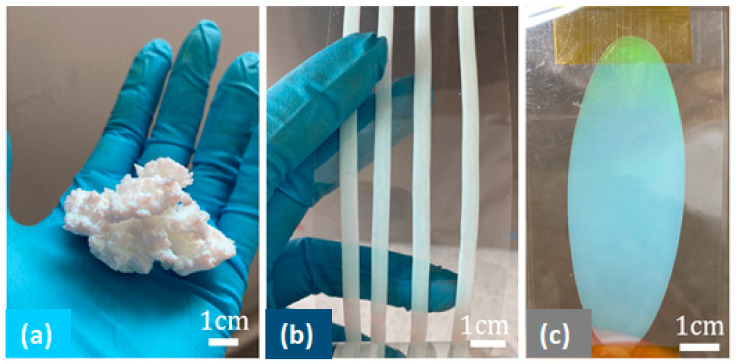
(**a**) Polymer opal BIOS precursor material, sedimented and dried from emulsion polymerization, which is melt extruded into (**b**) thin ribbons, 1 mm in thickness, which are laminated into (**c**) thin film polymer opals, which, prior to BIOS, have a milky color appearance due to inherent disorder.

‘Polymer opals’ (POs) are photonic crystals [1,2,3,4] generated from ensembles of core–interlayer–shell (CIS) polymeric particles. While polymer opals have been fabricated from a range of constituent materials [5], the study of these materials has focused mostly on spherical polystyrene (PS) nanoparticle cores coated with a softer poly(ethyl-acrylate) (PEA) shell [6,7,8]. In the synthesis of polymer opals, the PEA shell is grafted to the PS core by a thin layer of poly(methyl-methacrylate) (PMMA); a schematic of the resultant particles is shown in Figure 1a. This is achieved using a multi-stage emulsion polymerization technique, with a precursor product being obtained, following sedimentation and isolation (see Appendix A, Figure A1). This material is then dried and formed into ordered arrays using specifically engineered melt-shear techniques [9,10,11], which have evolved as the properties of POs have become better understood.

A simple representation of this process is shown in Figure 1b. While the notion of using shear to induce order in nanostructures is not new, what makes the polymer opal fabrication methods novel is their applicability to solvent-free granular systems and permanent ordering. Furthermore, in comparison to other methods of assisted self-assembly, PO fabrication methods result in structures that are not fully monolithic. In contrast to the majority of photonic crystals, POs have viscoelastic properties [12,13], resulting in reversibly stretch-tunable color appearance [14]. The small refractive index contrast (Δn = 0.12) between the PS core and PEA shell results in a Bragg diffraction giving intense structural color.

More generally, POs have a number of distinct advantages over other reported shear-ordered systems. Most of these systems are colloidal and, therefore, impermanent (unless set in resin), inherently changing the material and optical properties. POs are a rarity in shear-ordered photonics by virtue of being solvent-free systems, which removes the requirement for processing stages such as evaporation which are known to induce defects and cracking [15,16,17]. More broadly, roll-to-roll and shear techniques are of growing importance and interest within the field of functional thin films for optics and optoelectronics [18,19,20]; one of the main draws of POs is that they are fabricated using bespoke methods that exploit their robust end-rheology to address the challenges of mass production [21].

The manufacturing processes for polymer opals have been honed from the first ‘hot press’ compression molding method as developed by Ruhl and Hellmann [7]. This technique was developed in response to common observations in the fabrication of photonic crystals from drying and sedimentation [16], whereby thin layers would be prone to cracking [22]. Ruhl and Hellmann compress the PO precursor for one minute with a Collins press heated to 170 °C, which results in a melt flow of the polymer, perpendicular to the direction of pressure along the direction of the plates. As the particles are compressed, the PEA shells are distorted to fill the interstitial space between adjacent polystyrene nanoparticles. Pressures of up to 50 bar then force the cores to align along the wall of the press as the polymer spreads into a disc of approximately 100–300 µm thickness, resulting in crystallization at the edges of the PO. Ordering of the PS cores is made possible due to elastic forces of the compressed polymer chains within the shell material [6]. POs also display a so-called ‘sticky’ shell interaction, whereby, for two shells in contact, there is a short range attractive force [23]. This is countered by the hard-sphere repulsive interaction of polystyrene cores. As such, this work examines both new particle interactions and ordering methods.

Successful development of this method has involved a number of factors [24] pertaining to the material properties of the polymer nanoparticles. The particle distribution was required to be highly monodisperse, and the glass transition of materials was chosen such that cores had to remain rigid in order to crystallize (cross-linked polymers or silica being the most preferable materials), and these had to be encapsulated in an elastomeric shell that would be flexible enough to allow for deformation in order to fill the interstitials of the structure with compression. It was also required for the particles to be engineered such that the cores would not roam, necessitating a grafting interlayer, and that the shells were of a thickness suitable to allow some flow of the cores without compromising the grafting [25].

The next major development to the production of POs came with edge-induced rotational shear (EIRS) [26]. This method addresses the scalability issues of the hot press, and it was found to reproducibly result in reproducible bulk ordering. EIRS is a multi-stage process; firstly, the CIS nanoparticles are homogenized, melted, and extruded into ribbons of 1 mm thickness (Appendix A, Figure A1). These ribbons are subsequently melt-laminated between PET sheets to form a PET–PO–PET sandwich, with the resultant thin film then drawn repeatedly over a heated brass apex/edge at shear rates of in the region of 1 s^−1^, resulting in shear-ordered PS cores. Optical diffraction and microscopy demonstrate that this results in an fcc arrangement, with defects potentially resulting in hcp or rcp twinning. Finlayson et al. also showed that there is sharpening of transmittance and (both bright- and dark-field) reflectance peaks, as a function of the number of shear passes.

Bending-induced oscillatory shear (BIOS) came as a further iteration to the manufacture of POs and is currently regarded as the optimal processing method. This is a multi-stage technique encompassing principles of both compression molding and EIRS. The development of BIOS was led by Zhao et al. [27,28]; it led to the notion of shear-ordering based on rollers and, thus, the development of a roll-to-roll process [21]. An example of a BIOS-processed PO film is shown in Figure A1.

Zhao et al. showed that that their process is highly scalable and able to produce ordered films over lengths of the order of 100 s of meters. The POs are sheared up to 40–60 times to iteratively induce crystallization by random hcp structural ordering. This has the effect of improving the visual appearance of structural color from the films. Subsequent processing was shown to lead to a deterioration of the film structures and color appearance, in a similar manner to EIRS as described by Wong et al. [29].

### Synthesis Background

This report examines the synthesis of polymer opal nanoparticles; the successful optimization and control of a wide parameter space toward a ‘dial-in’ synthesis capability represent a non-trivial research challenge. The emulsion polymerization process has been investigated to determine how experimental parameters—temperature and agitation (stirring) rate—can control of the eventual size of the PS cores. Whilst the role of these parameters and their effect on particle size is widely understood for many types of polymerizations, previously there has been little investigation of relevant parameters in the synthesis of POs. Furthermore, there appear to be a number of important divergences from the majority of the literature (for example, the use of two surfactants). We quantify the effect of reaction temperature and agitation rate on particle size, with the wider objective of moving toward a dial-in process for polymer opals such that the desired color appearance of the processed opaline films, a function of particle size, can be readily obtainable as part of a wider industrial scale fabrication pipeline.

The literature on the synthesis of polymer opals comprises of a number of patents describing variations on the manufacturing process reported above [30]. Additional information pertaining to theoretical particle sizes from proprietary sources and industrial third parties was based on reports of a series of syntheses with varying concentrations of surfactant and the resultant particle sizes [6,30,31,32,33].

The polymer opal core–interlayer–shell (CIS) nanoparticles are synthesized by a multi-stage emulsion polymerization (EP). This is a type of chain-reaction polymerization [34], described by a reaction whereby the monomer reacts only with a ‘reactive center’. In the case of EP, this refers to the growth of polymer chains within protective micelles, suspended in an emulsion. These micelles are then collapsed so that the polymer nanoparticles can be collected. EP can be understood by Smith–Ewart theory [35].

This technique has a number of benefits over other polymerization methods [36] such as solution polymerization [37,38] and bulk polymerization [39]. EP allows for good thermal control of the reaction [40], is also a relatively fast technique [41], and allows a high yield for POs. It has also been described as more environmentally preferable than techniques which rely heavily on large volumes of potentially harmful solvents (solution polymerization) [42]. EP allows for good control of particle size, unlike suspension polymerization, for example [43]. Perhaps the most attractive property of the process for polymer opal synthesis is that EP results in a low polydispersity [41,44,45]. The resultant particle distribution from EP depends on several interconnected factors, including but not limited to reaction temperature and thermal control, monomer purity, initiator selection and quantity, and agitation (stirring) rate.

For PO nanoparticle production, the EP procedure is a multi-step process encompassing two sub-types of reactions. The growth of the polystyrene cores is semi-continuous, where the rate of monomer addition into the vessel is highly controlled. The subsequent stages of PO nanoparticle growth (interlayer and shell addition) are re-initiated ‘seeded’ reactions [30]. Anderson and Daniels described the primary benefits of seeded reactions being a high degree of control over particle size and quantity, and that seeded reactions can be applied to reproducibly grow particles of a chosen diameter with high monodispersity.

## 2. Materials and Methods

The composite nanoparticle synthesis procedure is reported for 1 kg solid polymer yield. Variations on this method have been documented within the patent record [30] and elsewhere [7,46]. Figure 1c shows the apparatus configuration for synthesis, with the three main components shown from left to right: the reaction vessel, which is fed by peristaltic pump, from a secondary vessel containing monomer solution with additional surfactant. The diagram also demonstrates a water-cooled condenser, inlets for nitrogen in order to blanket the reaction from oxygen, which terminates the polymer chains, and an air outlet, which passively stabilized the internal pressure of the monomer vessel.

### 2.1. Synthesis Procedure

The materials (and suppliers) used are as follows: styrene, stabilized (Sigma Aldrich, St Louis, MO, USA), NaOH (Sigma Aldrich), MgSO_4_, dried (VWR, Radnor, PA, USA), 1,4-butanediol diacrylate [BDDA] (Sigma Aldrich, tech. grade, ~75 ppm inhibitor), sodium bisulfate (Acros Organics, Waltham, MA, USA), potassium hydroxide (VWR), Dowfax 2A1, 50% in water (Dow Chemical Company, Midland, MI, USA), ethyl acrylate (Acros Organics), allyl methacrylate (Acros Organics, 98% stabilized), iso-butyl methacrylate (Acros Organics, 99% stabilized), 2-hydroxyl methacrylate (Sigma Aldrich), methanol (2 L, VWR), acetone (VWR), and industrial methylated spirit [IMS] (VWR). The procedure for CIS nanoparticle synthesis was undertaken as detailed in Section 2.1.1, Section 2.1.2, Section 2.1.3, Section 2.1.4 and Section 2.1.5.

#### 2.1.1. Washing

Styrene was first washed with a cold solution of caustic soda (prepared from NaOH, 100 g to 1 L water). Each wash was repeated three times to remove stabilizers, where the volume of styrene was washed with equal volume of caustic soda. Following the titration, magnesium sulfate (MgSO_4_) was used as a desiccant, and waste solids were then removed using a 100 µm filter. These protocols followed earlier preliminary trials of styrene purification to remove any residual stabilizer products. The polydispersity indices of the final core–shell nanoparticles are shown in ESI (Table A1), for the cases with no purification, with deionized water washing, and with caustic soda titration and MgSO_4_ drying.

#### 2.1.2. Polystyrene Cores

The reaction vessel was nitrogen-flushed with a reflux condenser. The vessel was heated to and held at a temperature of 65 °C, stirred at 200–250 rpm. The reaction vessel was charged with demineralized water (1120 g), styrene (14.4 g), 1,4-butanediol diacrylate [BDDA] (1.6 g), and emulsifier (1.17 g). Immediately following this, demineralized water (12 g), initiator (2.07 g), and sodium bisulfate (0.29 g) were also added to the reaction vessel. The vessel was stirred for 10 min. Charged into the secondary vessel was de-mineralized water (360 g), styrene (280 g), BDDA (28 g), emulsifier (0.97 g), potassium hydroxide (1.6 g), and Dowfax 2A1 (0.88 g). This was stirred and simultaneously added dropwise to the primary reaction vessel at a rate of 10 g of solution per minute. Following completion of the addition, the reaction vessel was stirred for 10 min. This stage synthesized cross-linked polystyrene cores. Charged into the reaction vessel was demineralized water (2 g) and 0.1 g initiator (0.1 g). This was stirred for 10 min, re-initiating the reaction of the polystyrene particles.

#### 2.1.3. Interlayer, Shell Addition, and Particle Isolation

Charged to the secondary vessel was demineralized water (128 g), ethyl acrylate (100 g), allyl methacrylate (12 g), emulsifier (0.21 g), and Dowfax 50% 2A1 (0.84 g). This was added dropwise to the main vessel at a rate of 10 g of solution per minute. Following completion of the addition, the vessel was stirred for 30 min. This served to grow the poly(methyl-methacrylate) interlayer onto the polystyrene particles, which acted as seeds for further work (see below). To the secondary vessel, demineralized water (640 g), ethyl acrylate (402.4 g), iso-butyl methacrylate (140 g), 2-hydroxyl methacrylate (16.8 g), emulsifier (1.7 g), and potassium hydroxide (0.8 g) were added. This was then added dropwise to the primary vessel at a rate of 10 g of solution per minute and stirred for 30 min. This stage grafts the poly(ethyl-acrylate) shell onto the polystyrene/poly(methyl-methacrylate) core–interlayer particles which act as seeds. The solution was cooled, and solids were filtered using a 100 µm sieve and removed. To the filtered solution, methanol (2 L, VWR) and 20 mL of concentrated brine solution were added and stirred for 30 min. Next, an equal volume of demineralized water was added to the solution, which was then filtered using a 100 µm sieve. After rinsing with acetone and industrial methylated spirit (IMS), the solids were dried in a warm vacuum oven at 45 °C for 3 days.

#### 2.1.4. Control and Optimization of Reaction Parameters; Methodology

The aim of process optimization was to move toward PO synthesis as a ‘dial-in’ process, providing reproducible particle sizes of a high monodispersity. A deeper understanding of the critical experimental factors relevant to the growth process was required. Much of the reported work in this area has focused on the modification of surfactant quantity in particle size, and this is well understood for PO systems. However, given the proven importance of process temperature, and the role of agitation rate reported elsewhere [47] but not yet reported for polymer opal systems, it was decided that optimization studies should focus primarily on these variables. This would be carried out for a set surfactant quantity, and both the agreement with theory and the reproducibility would be examined. It is also well known that vessel volumes can have a significant effect on particle growth due to heat loss; hence, it was considered that a small-volume vessel (giving fixed polymer material masses of 100 g) would allow for the best process control.

With temperatures of 75 °C (and upward) having been previously employed, little study had taken place into low-temperature processes for POs synthesis. This is despite the preference of industry in most instances to use lower processing temperatures where possible for reasons of both process control and safety. The majority of the literature pertaining to the polymerization of styrene employs temperatures of 60 °C and upward, although there are reports of polymerization at temperatures as low as 50 °C with sodium dodecyl sulfate (SDS) surfactant [48,49]. Temperatures beyond 70 °C are rarely reported, as stability and rate control issues (such exothermic reactions risk a ‘runaway’ effect) become challenging beyond this. For this reason, the core growth was studied at 50 °C, 60 °C, and 70 °C, using agitation rates of 150, 250, and 350 rpm. Nomura et al. [50] reported on what they term ‘an optimum range of stirring speed’, and that this affects particle size and monomer conversion in a number of ways. Firstly, stirring affects the absorption of any oxygen in the atmosphere into the reaction emulsion. Secondly, particles agglomerate due to the agitation of the emulsion. Lastly, stirring causes the absorption of the surfactant into the monomer droplets, which serves to reduce the number of micelles in solution. The formation of a micelle is dependent on two factors: firstly, that the ratio of components (e.g., monomer, water, surfactant) is maintained within a viable range; secondly, that the absolute concentration of surfactant is maintained above the critical micelle concentration (CMC) limit. This work, and that of others [51,52], points to a ‘sweet spot’ of agitation rate, where monomer conversion is maximized. The stirrer used was a magnetic chaser bar of 3.5 ± 0.05 cm length with a diameter of 1.5 ± 0.05 cm, with a mass of 17.3 ± 0.05 g in conjunction with a VWR VMS-C4 stirrer hotplate. The reaction vessel was a 500 mL round-bottomed flask with a starting liquid volume of approximately 115 mL, which sat in a 500 mL heat-on block to ensure uniformity in heating.

#### 2.1.5. Synthesis Note

The quantities of emulsifier should be scaled with regard to desired particle size. Chern [10] outlines the importance of emulsifier in affecting the number of micelles in an emulsion, with reference to the CMC (the concentration of emulsifier required for micelles to form), and explains that this should be carefully tuned to alter the number of particles and, hence, particle size. This is the manner in which EP can be used to obtain different particle sizes and, thus, a broad range of structural color effects in polymer opals, with the standard range of EP PO particle diameters being around 150–350 nm. Particles of 164 nm total diameter, theoretically giving UV structural color, and particles of 336 nm display near-infrared structural color [6,30].

### 2.2. Particle Size Determination

#### 2.2.1. Dynamic Light Scattering

Dynamic light scattering (DLS) was used to monitor particle growth in near real time where adjusting reaction parameters was necessary to obtain a desired particle size. The Malvern Zetasizer Nano ZS (Malvern, UK), using ‘noninvasive backscatter’ (NIBS) methods, was employed for particle size and distribution analysis. First, 1 mL of latex was removed from the reaction vessel by burette, and dispersed in 100 mL of de-ionized water. This solution was agitated by hand, and 4 mL of this solution was placed into a transparent disposable polystyrene cuvette.

The cuvette was then encapsulated by a thermally insulated cover before being loaded into the instrument. Prior to the beginning of measurement, the suspension was allowed to equilibrate to 25 °C over the course of 60–90 s. The measurements provide three averages of particle diameter, Z-average, and particle size polydispersity index. Following measurement, the average of each of these was recorded with outliers rejected. During synthesis, the particle size was measured every 15–30 min in order to observe growth rate, and then half an hour following completion of the monomer addition.

In this paper, all reports of polydispersity index (PDI) involve a dimensionless measure referring to the broadness of the size distribution. Thus, PDI can be extrapolated from the autocorrelation function in DLS. Values range from 0 (monodisperse) to 1 within the Zetasizer software v3.30. On a final note, given the in situ nature of our methodology, DLS reliably provides the all the crucial information about size and size distribution of particles in a real-time fashion, in a way that electron microscopy (EM) analysis usefully cannot.

#### 2.2.2. Electron Microscopy

The final synthetic batch products may be re-dispersed into excess ethanol by ultra-sonication, so that individual particles may be deposited and dried onto suitable substrates for imaging with a scanning electron microscope (SEM). For SEM, samples were pre-coated with around 4 nm of Pt/Pd, to reduce charging effects. Measurements were taken with the JEOL 840A cryo-SEM instrument (Akishima, Japan) at room temperature, operating in secondary electron detection (SED) mode at 10 kV accelerating voltage.

## 3. Results and Discussion

### 3.1. Low-Temperature Synthesis

**Figure 2:**
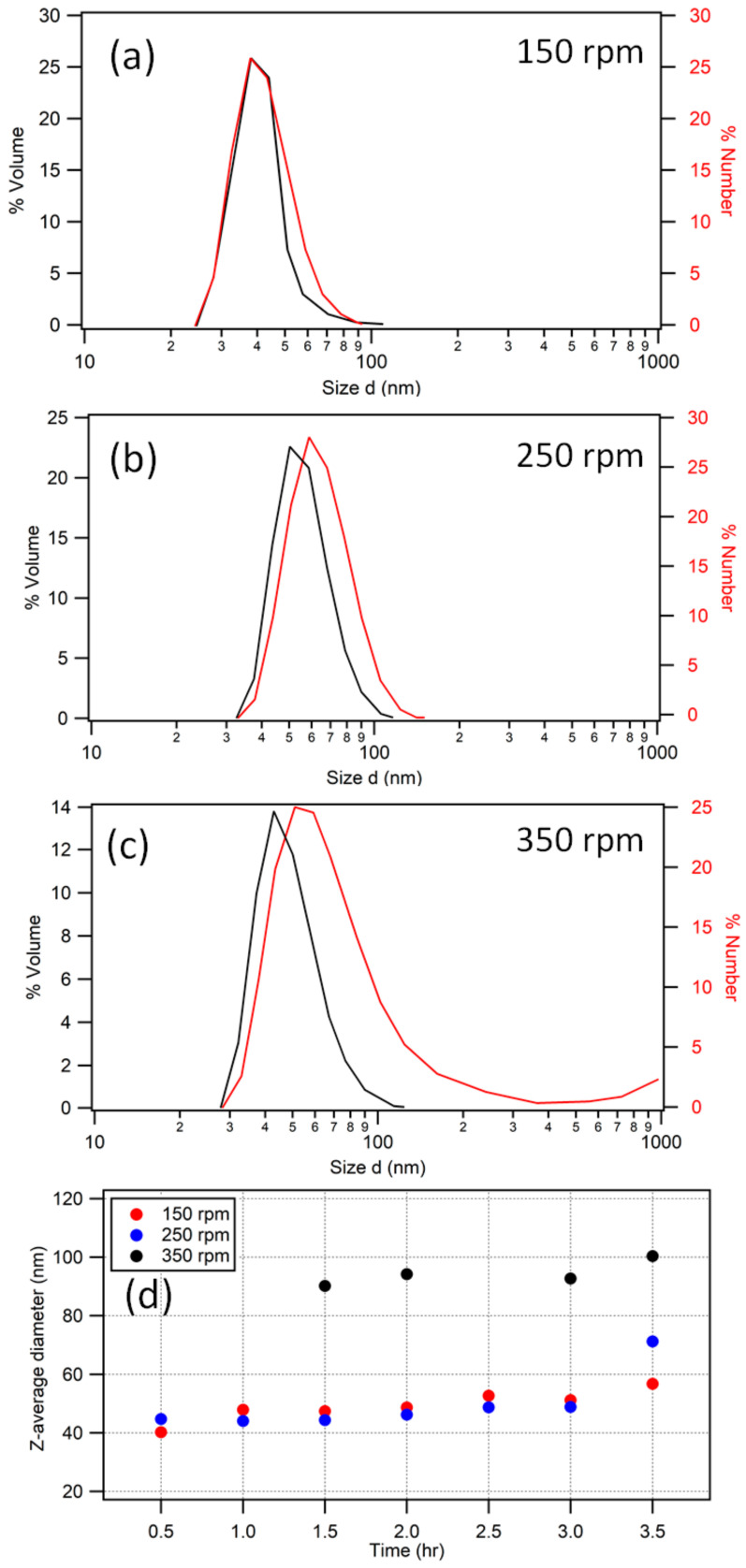
(**a**–**c**) The distribution of polystyrene nanoparticles processed at 50 °C for 3.5 h (end) in terms of both volume (black curves) and number (red curves) for 150, 250, and 350 rpm agitation rates. At 350 rpm, a secondary population of large particles is evident; however, from number analysis, this is seen to comprise only a small fraction of the particle content. (**d**) The z-average-based particle diameters for 150, 250, and 350 rpm measured over time. Points for 350 rpm at early times are out of range due to populations of larger particles.

At 50 °C, for each agitation rate, there was significant styrene monomer remaining, as evidenced by a phase separation in the reaction vessel. The monomer was estimated as a volume percentage of the vessel content to be 15% for 150 rpm, 10% for 250 rpm, and <5% for 350 rpm. Previously, the synthesis of polystyrene nanoparticles has been carried out at lower temperatures with a good degree of monodispersity [53]; however, this is often over longer time periods in excess of 24 h (and often as a batch emulsion polymerization). This is in contrast to the polymer opal synthesis method, which calls for a semi-continuous emulsion lasting up to 4 h for the core synthesis [5,6,7]. DLS measurement (Figure 2) of the particle sizes concurred with the above literature in showing that the conversion of styrene at 50 °C was very slow.

Table 1 reports the PDI of the nanoparticles throughout the reaction, with polydispersity seen to increase with stirring rate. With PDI averages in the region of 0.1–0.2, these solutions displayed a high degree of polydispersity compared to that usually expected for the synthesis. Due to the presence of multiple populations as evidenced by light scattering, Z-average is an unreliable metric (because it is based on the assumption of a single monodisperse population of spherical particles). Although an increased stirring rate increased the final particle size (Figure 2d), evidencing an improvement in monomer conversion, it also resulted in more secondary populations contributing to these increased polydispersity; for 350 rpm, there were up to three distinct populations of particle sizes, and large particles of ~3.5 µm appeared in each synthesis at various stages (see Appendix B, Table A2).

Of note, in the data for 150 and 250 rpm, there are significant PDI fluctuations from 0.5 h to the end. By contrast, at 350 rpm, the PDI values show a smooth decrease in values, perhaps indicative of a more heterogeneous distribution of monomer. Considering the quantity of unreacted styrene on the surface, together with the difference in expected and measured particle size, it is also reasonable to suggest that there were large monomer droplets in the emulsion, which may have introduced errors into the measurement of polydispersity.

#### Comparisons with the Literature

Nomura et al. [50] explained that, when the monomer phase is separated from the emulsion, the concentration of monomer is not consistent throughout the reaction vessel and causes difficulties in accurately sizing the particles. This effect may be particularly pertinent at low temperatures because of the lower solubility of styrene [54] and would further support the idea that the high PDI is a consequence of unreacted monomer. Figure 2d also allows a number of observations to be made with reference to particle growth rate and size; for an agitation rate of 150 rpm, growth is very limited, with a final particle diameter of only 56.9 ± 0.5 nm. This is partly a consequence of the poor mixing of the styrene with the emulsion, as discussed by Roudsari et al. [51], who also identified this issue. Nomura et al. investigated slower agitation rates, and concluded that the main mechanism driving particle growth involves monomer seepage from the droplet phase as opposed to agglomeration occurring with high stirring speeds. At a 250 rpm agitation rate (Figure 2d), growth of the particles was again very slow, until the point at which the addition was completed, where the particles were seen to grow suddenly by more than 20 nm. While a slow rate of growth is one hallmark of oxygen termination, this is judged unlikely according to the lack of stunting observed in repeats of the syntheses. In other emulsion polymerization systems at lower reaction temperatures, longer ‘induction times’ associated with slower agitation rates have been reported [51], and the pattern of particle growth seen across the three different agitation rates is more in agreement with this theory.

The 350 rpm synthesis gave a (primary) population of around 120 ± 0.5 nm. In agreement with the literature, the improvement in mixing evidently contributed to the increased miscibility of styrene with water. Therefore, a greater particle size was achieved as the styrene was better distributed throughout the emulsion. It is also known that faster agitation can increase the particle size on account of the agglomeration of particles in emulsion from shear. As reported by Roudsari et al. [55], the shear forces within a fast-flowing emulsion also has the effect of deforming the monomer droplets, which can result in irregularity in the movement of monomer through the emulsion. This may explain the evolution of secondary populations in the low-temperature systems, where the rate of polymerization was not so fast as to consume the monomer following droplet breakup.

The low-temperature polymerization of styrene highlights the role of chemical and physical factors contributing to a number of effects on particle size and polydispersity. Despite the observation of polymerization at low temperatures, PDI generally remains too high to be suitable for further synthesis of polymer opal interlayers and shells. However, this process could potentially be adapted for the growth of very small particle cores on the order of 50–70 nm. This could be achieved by the addition of only a small quantity of monomer for 0.5–1 h, as opposed to the procedural 3 h addition, considering the PDI of <0.1 at these timescales. Clearly, this would require adjustment of reactant quantities for the stages of interlayer and shell formation that follow core growth, thereby ensuring the necessary relative volume of CIS particle layers.

### 3.2. Higher-Temperature Synthesis

Many of the issues observed for 50 °C were not present for higher-temperature synthesis. Although trace quantities of monomer remained in solution, at 60 °C and above, there was no evidence of monomer collecting on the surface of the emulsion. This is indicative of better homogeneity of emulsion mixing due to the improved miscibility of styrene in water, and it also tends to an improvement in the reproducibility of particle sizing due to the concentration of monomer and, therefore, polymerized particles throughout the vessel being more consistent. Higher temperatures resulted in faster reaction rates, which would also contribute to the higher conversion rate of monomer over the timescales of the syntheses. Consequently, this has the effect of preventing secondary populations of particles occurring. Even at the highest agitation rates, where there is potential for monomer droplet deformation and breakage, the higher temperatures likely enable stray monomer to be consumed more quickly. As such, the nanoparticles from the higher temperature syntheses were significantly more monodisperse than those obtained for 50 °C synthesis (with PDIs reported in Table 2). As seen previously, PDI was not seen to track with reaction time, and there was additionally little measurable difference in PDI between 60 and 70 °C. The low PDIs resulted in good agreement between Z-average and intensity scattering size determinations.

#### Comparisons with Lower-Temperature (50 °C) Syntheses

**Figure 3:**
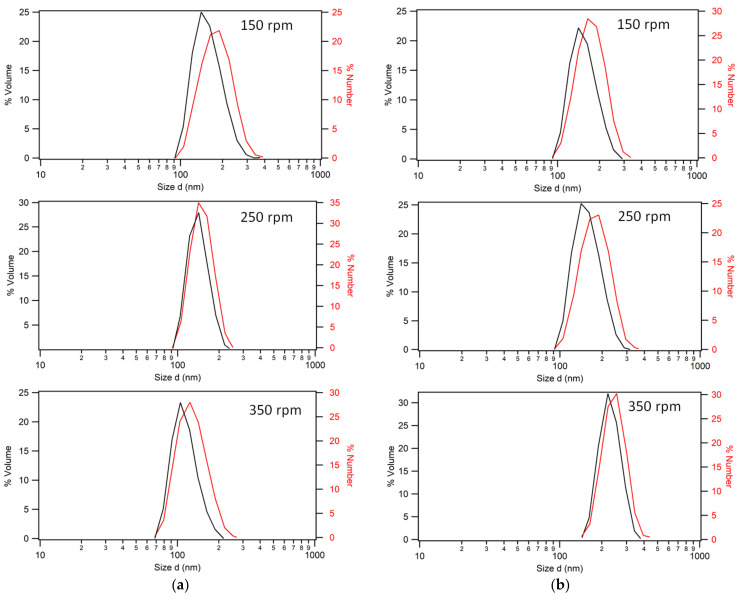
(**a**) Dynamic light scattering (DLS) of polystyrene nanoparticles synthesized at 60 °C, where the stir rate is shown to have the effect of decreasing the size of the most predominant population. (**b**) Comparative data for polystyrene nanoparticles synthesized at 70 °C with stir rates of 150, 250, and 350 rpm, as indicated.

In line with the low-temperature synthesis results, stir rate was seen to have a discernable effect on the final particle size population. For 60 °C, this was empirically seen to follow an inverse relationship with stir rate, with 150 rpm agitation resulting in larger particles of approximately 170 ± 0.5 nm diameter compared to 125 nm particles gained from 350 rpm agitation. Evidently, the agglomeration causing larger particles for 50 °C is not significant for this higher process temperature. This could be due to the accelerated thermal decomposition of the initiator at 60 °C as compared to 50 °C, leading to free radicals which are then better dispersed at higher temperatures, and which react faster due to the shorter induction times. The final particle size distribution at each agitation rate appears to correlate with stir rate in Figure 3a.

However, the opposite was seen for the 70 °C syntheses, shown in Figure 3b: 240 ± 0.5 nm particles from 350 rpm and 160 nm from 150 rpm. This is a discrepancy worthy of note because previous work [47] referred to an optimum stir rate (usually 200–250 rpm) in terms of monomer conversion. Furthermore, when this rate was exceeded, there was a corresponding decrease in particle size. It is not clear why this relationship exists, and investigation into the complex mechanisms at play is beyond the scope of this work. It should be acknowledged that particle growth rate is related to the increase in volume, which goes as a function of the radius cubed. Hence, future work in this area should consider the seed particle stage of the reaction, as well as the initiation stages prior to the dropwise addition of monomer, because calculation of particle number and concentration would be highly beneficial to modeling of the theoretical particle sizes obtained for complete styrene conversion.

### 3.3. Reaction Dynamics and Deviation from Emulsion Polymerization Conditions

A notable finding of this work is a significant divergence from the expected relationship between final particle size and temperature. Emulsion polymerization reports indicate a very well understood, repeatable link between temperature and final particle size, with higher temperatures reported to result in smaller particles across a wide range of reaction systems [48,56,57,58]. One major contributing reason for this is that, during the Initiation stage, there is faster thermal decomposition of initiator into free radicals which results in more particle growth sites within the emulsion, thus causing smaller particles on average [59]. This was not seen with this work, where larger particles resulted from higher processing temperatures for each agitation rate that was investigated. One potential explanation for this could be lack of reaction completeness; however, as shown from high-performance liquid chromatography analysis (HPLC) in Appendix B, Table A3, these syntheses show a high degree of monomer conversion—usually around 99.5%. Previous studies on EP did not adequately encompass the specific complexities of PO synthesis, such as the need for two surfactants and the use of co-monomers such as 1,4-butanediol diacrylate (BDDA). However, these results from our investigation have similarities to dispersion polymerization (DP) processes, where particle size is also seen to increase as a function of temperature [60]. These results, furthermore, show consistency with DP resulting in fast monomer conversion at higher temperatures [61,62].

Barrett [62] explained how dispersion polymerization varies from emulsion polymerization as a result of how polymer chains combine with existing particles, as opposed to emulsion polymerization, where the particle initiation stage is the primary determining factor in quantity of particles and, thus, final size. Another characteristic factor of DP is that the higher temperatures lead to more of the free radicals successfully resulting in polymerization and, hence, more chains being absorbed to form larger particles [63]. Hence, while emulsion and dispersion polymerization share some similarities, the differences in reaction mechanisms between the two systems result in very different dynamics, and it is hypothesized that there is a critical point at some stage in the addition of the monomer that the emulsion instead becomes a dispersion.

**Table 2:** The PDIs (±0.0005) of syntheses for stir rates of 150, 250, and 350 rpm at 60 and 70 °C as measured throughout the reactions. The end measurements were taken for 30 min following the completion of the monomer addition. The end time was at 3.5 h.

Time (h)	60 °C			70 °C		
	150 rpm	250 rpm	350 rpm	150 rpm	250 rpm	350 rpm
0.5	0.003	0.031	0.022	0.02 ^†^	0.010	0.018
1.0	0.016	0.010	0.011	0.017	0.008	0.017
1.5	0.015	0.015	0.020	0.015	0.019	0.041
2.0	0.013	0.009	0.019	0.019	0.017	0.041
2.5	0.037	0.018	0.008	0.009	0.010	0.029
3.0	0.024	0.016	0.020	0.021	0.033	0.017
End	0.013	0.010	0.012	0.019	0.026	0.029

^†^ Estimated value.

**Figure A2:**
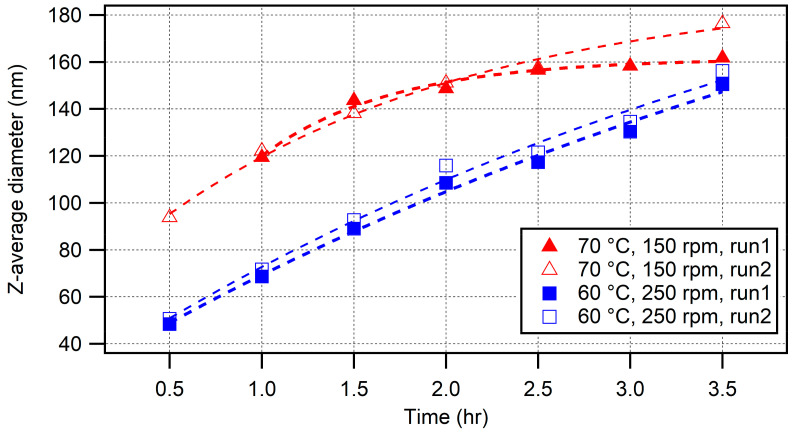
Repeat syntheses of the polystyrene nanoparticles at 60 °C and 70 °C (stir rate 250 and 150 rpm respectively), showing good agreement in final particle size and implied rate of monomer conversion.

This critical point potentially occurs around 2 hr into the reaction, where changes are seen to occur in the relationship between stirring rate and particle size, as observed at both 60 °C (250 to 350 rpm) and 70 °C (150 to 250 rpm). This notion has a mathematical and physical basis when the growth procedure is considered; at the initiator stage, BDDA comprises 0.14% of the total reaction content by mass. This is compared to a significantly larger 4.2% of the drop-fed monomer, which following completion of the addition, brings the total BDDA concentration of the reaction to 1.6%. Hence, one explanation for the relationship of particle size with temperature is that the increasing concentration of BDDA in the emulsion at some point becomes sufficient enough to result in a dispersive system, perhaps under some conditions where the BDDA is preferentially polymerized [64] over styrene. The time stage at which this subtle transition occurs may vary depending on the reaction conditions such as temperature and agitation rate, which affect the homogeneity of the reaction. Further study would be required to confirm this finding, and to find out how this hypothesis would hold under examination over a wider temperature range, with greater consideration also given to the role of the two anionic surfactants in use. This work was found to be highly reproducible over multiple runs to a good level of agreement, as shown in Appendix A Figure A2, which shows the particle growth over sets of repeat syntheses. However, further repetition of these investigations under a wider range of conditions may also be useful.

### 3.4. Final Particle Shape Integrity and Sizing

**Figure 4:**
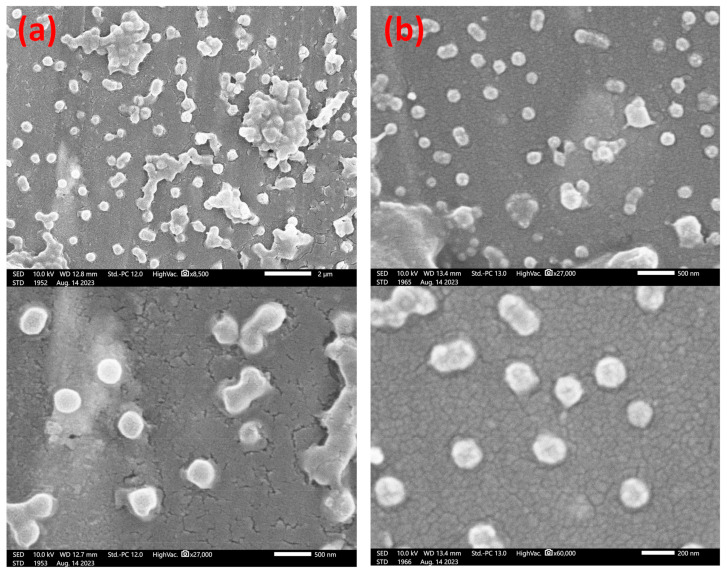
SEM images of spherical core–interlayer–shell polymer opal precursor particles, as synthesized in this work. In (**a**), larger particles at the near-IR end of the structural color range are shown, in comparison to smaller particles for the UV–blue wavelengths in (**b**). Indicative scale bars in (**a**) are 2 μm (**top**) and 500 nm (**bottom**); scale bars in (**b**) are 500 nm (**top**) and 200 nm (**bottom**). Using Image-J analysis software (version 1.54d), the measured particle diameters from these images were 351 (±22) nm and 178 (±14) nm respectively.

Whilst there are practical limitations in providing electron microscopy images of the synthesizing particles ex situ at present, it is nonetheless possible to analyze the end CIS particles, as developed from the batch core materials. SEM images from key examples of our syntheses are shown in Figure 4, comparing the case of particles grown from small (ca. 100 nm diameter) cores with those developed from a typical synthesis endpoint (ca. 300 nm diameter).These illustrate the successful control of particle (spherical) shape integrity and polydispersity at the beginning and endpoint of core growth, strongly confirming the observations reported in the literature reported by the authors [65,66] and by others [9,25], as well as the overall robustness of the synthesis methodology. The measured PDI, as inferred from the standard deviation in sizes, was in the range of 0.05 to 0.10 in these analyses, which was slightly above that as determined by DLS; however, there were far fewer counted particles (*n* < 50), giving a likely higher statistical error.

### 3.5. Toward Polymer Opal Synthesis as a ‘Dial-In’ Technique

**Figure 5:**
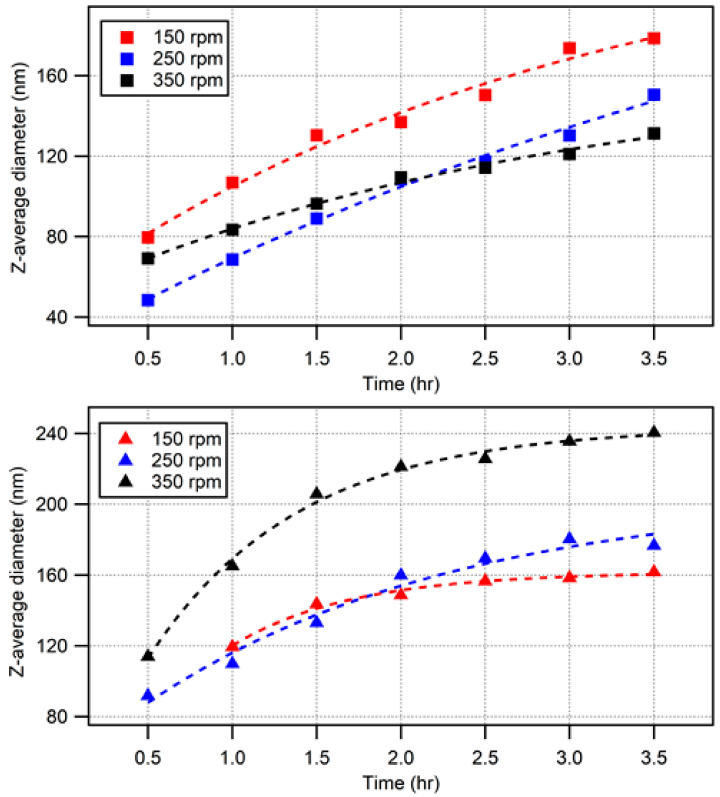
The Z-average based particle size for 60 °C ■ and 70 °C ▲ syntheses as monitored in line over the time of the monomer addition, with final particle size indicated at 3.5 h.

In moving toward a dial-in process, it is desirable to understand how the required characteristics of the PO cores can be obtained (in terms of size, polydispersity, number, or volume). This research demonstrates how it is possible to grow PO cores under a range of conditions with multiple routes to obtaining a given particle size. An overview of this is given in Table 3, where the time taken to obtain the core size need for the specified resultant color appearance as indicated by the literature is reported [66,67,68]. This is as determined from Figure 5, where these times are extrapolated from the exponential fit curves. Temperatures of 60 and 70 °C are considered attractive and viable because all syntheses under these conditions gave an excellent level of monodispersity suitable for interlayer and shell growth. This work follows the observation-based assumption of all polymerization being complete, as discussed above.

**Figure 6:**
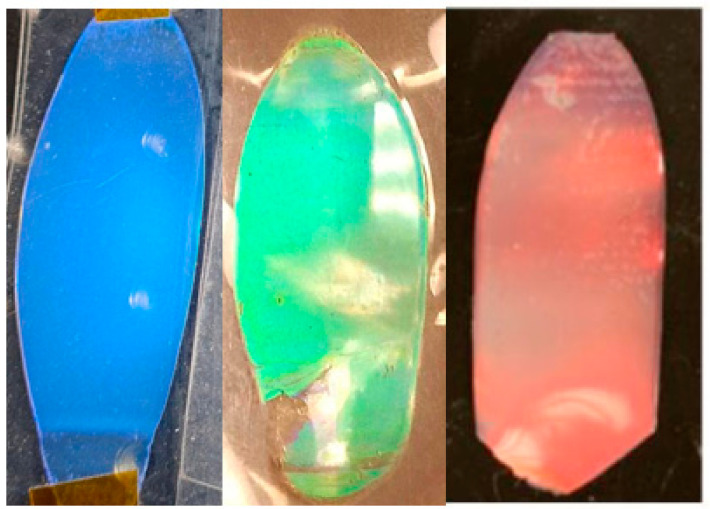
Photographic images in reflected white light showing polymer opal films generated by the BIOS method from the solid batch materials at the end of syntheses, according to the literature protocols. From left to right, the structural colors are blue (core/CIS diameters ≈ 139/184 nm, $\lambda_{0}$ = 463 nm), green (core/CIS diameters ≈ 175/230 nm, $\lambda_{0}$ = 549 nm), and deep red (core/CIS diameters ≈ 230/330 nm, as viewed at ≈30° from normal incidence,${\;\lambda }_{0}$ = 715 nm).

The generation of film samples from the intermediate aliquots taken from the reaction vessel is not feasible, as the process requires a minimum of ~1 g of dried batch material for reliable extrusion and production. However, we can nonetheless report the color quality of the BIOS films, as developed onward from the endpoint of syntheses. Representative examples are shown in Figure 6, illustrating how the measured core and CIS diameters result in peak reflectance values ($\lambda_{0}$), which place the visibly apparent structural color characteristics well within the target spectral ranges implied in Table 3.

This work represents the detailed evaluation of particle size distribution metrics of polymer opal synthesis, studying the role of temperature and agitation rate in a previously unreported level of detail. There is discussion of these reaction parameters and their effect on particle size and resultant polydispersity, as well as how this maps to the theorized particle sizes from existing literature. New insights are also reported in terms of the behavior of the reaction, which, under some conditions, more closely matches that of a dispersion rather than an emulsion polymerization, with some hypotheses as to why this occurs in these types of under-reported syntheses of polymer nanoparticles. Further work in the study of PO synthesis should give greater credence to examination of monomer conversion rate, with techniques such as HPLC for example. Probing of the initial reaction stages, particle seeding, and initiation would be highly beneficial toward understanding the concentration of particles and number of growth sites, alongside the investigation into reaction completeness. Essential to any industrial application, there is a distinct requirement for research into opal synthesis to turn toward larger volumes of 10 s or even 100 s of liters. This should be coupled with study into reproducibility of these syntheses for larger vessels and varying quantities of surfactant, as well as a finer control over temperature, agitation rate, and study of different stirring geometries.

## 4. Conclusions

This report examines the synthesis of the base core–interlayer–shell nanoparticles for polymer opals. A fully engineered control of the eventual size and monodispersity of the polystyrene (PS) cores, along with control of a wide synthesis parameter space, represents a non-trivial research challenge. We examined the role of key experimental parameters in the establishment of viable and deterministic synthesis conditions, by optimizing the temperature, reactant purity, and agitation (stirring) rate.

On the laboratory processing scale reported here, the synthesis of polymer opal cores is seen to conclusively result in highly monodisperse polystyrene cores (polydispersity index < 0.02) when processed at temperatures of 60 °C and 70 °C, with resultant particle size seen to be highly tunable as a function of agitation rate and temperature. A stirring rate of 250 rpm was seen to be optimal among those examined, in close agreement with the literature, resulting in good dispersal of surfactant and monomer while minimizing the potential for adverse polydispersity.

A notable finding of this work, and of a more fundamental interest, is a significant divergence from the expected relationship between final particle size and temperature from emulsion polymerization. We expect this to be the focus of further investigation, both experimental and theoretical, as to the effects of vessel homogeneity and the potential for dispersive polymerization reaction regimes to develop.

A clear roadmap is presented for a dependable ‘dial-in’ approach to obtaining particle sizes corresponding to a target structural color property in the end product, which we demonstrate can span between the ultraviolet and near-infrared regions of the spectrum. Our approach, thus, facilitates application-driven fabrication pipelines for these versatile polymeric photonic materials.

## Figures and Tables

**Table 1:** The polydispersity indices (±0.0005) of polystyrene cores synthesized at 50 °C for stir rates of 150, 250, and 350 rpm. The average of three runs is reported for each measurement, with the ‘end result’ having been a sample taken 30 min following the termination of monomer addition. The end time was at 3.5 h.

Time (h)	150 rpm	250 rpm	350 rpm
0.5	0.047	0.025	1.000
1.0	0.122	0.139	0.688
1.5	0.032	0.147	0.380
2.0	0.131	0.149	0.348
2.5	0.206	0.215	0.3 ^†^
3.0	0.103	0.194	0.228
End	0.111	0.017	0.238

^†^ Estimated value.

**Table 3:** The time taken (h, ±0.2) to synthesize polystyrene cores of sizes corresponding to the final color appearance obtained from the PO films, assuming growth of the interlayer and shell as per reported protocols. No synthesis was continued beyond the endpoint of 3.5 h.

Structural Colour, Core Size (nm)	60 °C			70 °C		
	150 rpm	250 rpm	350 rpm	150 rpm	250 rpm	350 rpm
UV, 113	1.1	2.1	2.1	0.8 *	0.8	-
Violet, 126	1.5	2.7	3.4	1.1	1.2	0.6
Blue, 151	2.3	3.5	-	1.9	1.8	0.8
Blue-green, 167	3.1	-	-	-	2.7	1.0
Green, 175	3.4	-	-	-	3.1	1.1
Yellow, 188	-	-	-	-	-	1.3
Orange, 199	-	-	-	-	-	1.5
Red, 207	-	-	-	-	-	1.7
Infrared, 231	-	-	-	-	-	2.9

* This represents an empirical upper limit to the time taken to reach this size.

## Data Availability

Data can be accessed from the Aberystwyth University PURE repository. https://doi.org/10.20391/0e616bb6-503c-4e64-9233-64c593ef3082.

## Appendix A

Supporting figures.

## Appendix B

Supporting tables.

**Table A1:** The polydispersity index, averaged across three runs, for core–interlayer–shell polymer opal particles, where the styrene for the opal cores is purified with a number of methods.

Styrene Purification Method	C–I–S Particle PDI (±0.0005)
None	0.074
Water only	0.054
Caustic soda + MgSO_4_	0.033

**Table A2:** Measured particle sizes (nm, ±0.5 nm) determined by the intensity of light scattering for stir rates of 150, 250, and 350 rpm at 0.5 h intervals at 50 °C. The primary and secondary populations are indicated as well as the percentage of scattered light intensity corresponding to each particle size. The end time was at 3.5 h.

Time (h)	150 rpm		250 rpm		350 rpm	
0.5	43.0	100%	46.9	100%	1674	67.1%
	-	-	-	-	61.5	30.1%
	-	-	-	-	1804	2.8%
1.0	49.1	100%	47.2	100%	84.8	81.4%
			-	-	77.6	17.2%
			-	-	1795	1.4%
1.5	49.8	100%	47.3	100%	101.1	86.8%
			-	-	3763	11.9%
			-	-	1795	1.3%
2.0	47.7	100%	49.1	99.6%		
			1781	0.4%		
			-	-		
2.5	52.8	98.2%	48.0	96.9%		
	3485	1.8%	5030	3.1%		
					
3.0	54.5	100%				
					
					
End	56.9	100%				

**Table A3:** High-performance liquid chromatography (HPLC) analysis of aliquots taken from the synthesis following completion of monomer addition and subsequent stirring. A high percentage of monomer conversion was observed across all reaction conditions.

Reaction Parameters	Unreacted Styrene Content (%) (±0.0005%)
60 °C, 150 rpm	0.501
60 °C, 250 rpm	0.655
60 °C, 350 rpm	0.104
70 °C, 150 rpm	1.412
70 °C, 350 rpm	0.481
